# Chromosome Copy Number Variation and Control in the Ciliate *Chilodonella uncinata*

**DOI:** 10.1371/journal.pone.0056413

**Published:** 2013-02-20

**Authors:** Kevin J. Spring, Stephanie Pham, Rebecca A. Zufall

**Affiliations:** Department of Biology and Biochemistry, University of Houston, Houston, Texas, United States of America; Institut de Genetique et Microbiologie, France

## Abstract

Copy number variations are widespread in eukaryotes. The unusual genome architecture of ciliates, in particular, with its process of amitosis in macronuclear division, provides a valuable model in which to study copy number variation. The current model of amitosis envisions stochastic distribution of macronuclear chromosomes during asexual reproduction. This suggests that amitosis is likely to result in high levels of copy number variation in ciliates, as dividing daughter cells can have variable copy numbers of chromosomes if chromosomal distribution during amitosis is a stochastic process. We examined chromosomal distribution during amitosis in *Chilodonella uncinata*, a ciliate with gene-size macronuclear chromosomes. We quantified 4 chromosomes in evolving populations of *C. uncinata* and modeled the amitotic distribution process. We found that macronuclear chromosomes differ in copy number from one another but that copy number does not change as expected under a stochastic process. The chromosome carrying SSU increased in copy number, which is consistent with selection to increase abundance; however, two other studied chromosomes displayed much lower than expected among-line variance. Our models suggest that balancing selection is sufficient to explain the observed maintenance of chromosome copy during asexual reproduction.

## Introduction

A major theme in eukaryotic genomics is the abundance of copy number variations (CNV) of genetic elements. CNV are differences in genome content between individuals in a population resulting from insertions, deletions, and duplications that range in size from 1-kb to megabases or even whole chromosomes [1]–[3]. CNV create genomic structural variation that can be detected by comparing individuals within a population [4]. Many CNV have been associated with various human diseases [1], [5]–[7]. CNV may also allow for adaptive evolution [8]–[9].

*Chilodonella uncinata* (Cl: Phyllopharyngea) is a ciliated microbial eukaryote whose unusual genome structure provides a unique opportunity in which to study CNV. Like all ciliates, *C. uncinata* has two functionally distinct genomes housed in the micronucleus (MIC) and macronucleus (MAC) [10]. Most transcription that results in protein production occurs in the MAC. The MIC is quiescent during vegetative growth but undergoes meiosis and is exchanged during conjugation [10]. During development, the macronuclear genome undergoes massive reorganization resulting in thousands of ‘gene-size’ chromosomes. Each chromosome is amplified up to thousands of times, making the MAC highly polyploid [10]–[12].

In addition to the DNA duplication and loss that happen in all eukaryotes, two unique processes also potentially contribute to copy number variation in *C. uncinata*. First, chromosomes may be amplified to different numbers during development in different cell lineages. In *Oxytricha*, another ciliate with gene-size chromosomes, chromosome copy number is controlled by parental RNA abundance during development [13]. Thus any differences in RNA abundance between lineages will result in CNV. The second process is changes in copy number during asexual division, which is the focus of the work reported here. During asexual division, the micronucleus undergoes conventional mitosis, but the MAC undergoes amitosis, a division in the absence of mitotic spindles and programmed segregation of chromosomes [10]. The MAC chromosomes are duplicated and distributed to the two daughter cells stochastically [14].

Current evidence suggests that ciliate species differ in their regulation of chromosome copy number. For example, *Tetrahymena thermophila*, which has large, multi-gene MAC chromosomes, regulates chromosome copy number such that every chromosome is maintained at a ploidy of ∼45 (except the rDNA containing chromosome, which is apparently highly amplified in all ciliates) indicating coordinated regulation of amplification and segregation [15]–[16]. In *Euplotes crassus,* which has gene-size chromosomes, DNA amplification is regulated individually for each chromosome and that number is apparently maintained during segregation [17]. However, Duerr and colleagues [14] conclude that data on senescence of ciliates with gene-sized chromosomes indicate that chromosome copy number is not regulated during asexual division. In this study, two models of MAC chromosome segregation were compared: the regulatory model and the stochastic model. The regulatory model assumes that chromosome copy number is returned to the parental state after each division except in the case that a chromosome is completely lost. The stochastic model assumes differences in copy number that occur during division are maintained. In each model, cell death occurs when at least one MAC chromosome is lost. By comparing the results of this model to the number of asexual divisions a lineage survives, Duerr et al. [14] conclude that the regulatory model cannot explain the observed cell line death in species with gene-sized chromosomes.

Here, we examine chromosome copy number in *C. uncinata*, a lineage of ciliates that have independently evolved gene-size chromosomes from those previously studied, i.e. *Oxytricha* and *Euplotes* (Cl: Spirotrichea) [11]. We first demonstrate that different chromosomes are present at different copy numbers from one another. We then follow the changes in chromosome copy number after many rounds of asexual division. We find that the mean chromosome copy numbers do not change except for the chromosome carrying the ribosomal RNA genes, and the variance in copy number between lineages changes less than predicted under stochastic segregation of chromosomes into daughter lineages. A model of stabilizing selection can account for at least some of the reduced variance; however, we cannot rule out the possibility of a molecular regulatory mechanism to reduce variance in chromosome numbers among daughter cells.

## Methods

### Cell Culture

The *C. uncinata* culture used in this study is from a population in Poland (ATCC® #PRA-257; described in [18]). All lineages are descendent from one single-cell clonal isolate from this culture. Cells were grown in Cereal Wheat Grass medium, a liquid medium containing one part cereal wheat grass (Scholar Chemistry) steeped in hot water for 1 hour and diluted with one part micron-filtered deionized water with 0.5% (w/v) Na_2_HPO_4_. One day prior to starting a new culture of *C. uncinata*, the medium was inoculated with *Klebsiella pneumoniae* and incubated at 30°C for 24 hours. Inoculating the bacterial food source prior to adding *C. uncinata* allows for a food rich medium, which reduces the chance of starvation and subsequent conjugation. The inoculated medium was aliquoted into 48-well culture trays in 0.5-ml portions. *C. uncinata* isolates were added to culture wells and incubated at room temperature in the dark. Cultures were visually inspected for conjugating pairs during growth of the cell lines and prior to starting a new culture; no evidence of conjugation was found throughout the experiment.

### Experimental Evolution

Sixty single-cell clonal lines were started from the original isolate culture. Cultures were maintained by transferring a single-cell from an exponential phase culture to new bacterized medium. A total of 23 single-cell transfers were made over the course of about 5 months. Transfers were made in duplicate and in the event of a cell line loss the duplicated line was subcultured in the next transfer step.

The number of rounds of asexual division that occurred between each transfer was estimated by a growth curve generated by counting the density of cells on a C-chip hemocytometer. Triplicate measurements were taken every 8 hours for 48 hours. The total number of generations that occurred between measurements is estimated by
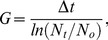
(1)where *N_o_* is the original cell density, *N_t_* is the cell density at time *t*, and *Δt* is the difference in time between measuring *N_t_* and *N_o_*. The total number of generations that occurred over the course of the experiment was calculated by multiplying *G* by the total time of the experiment. The total number of generations was found to be about 276, or an average of 12 generations between each subculture.

### DNA Extraction

Bulk cell cultures were grown for DNA extraction at the start and end of the experiment. We used extraction time points ∼276 generations apart to increase our power to detect changes in chromosome copy number without losing all of our cell lines. Cultures were transferred to 50-mL conical tubes containing 20-mL of bacterized Cereal Wheat Grass medium and incubated at room temperature for 5 days. *C. uncinata* inhabits mostly the surface and bottom of culturing containers so the conical tubes were set horizontally for maximum area of growth. Cultures for DNA extraction were grown in three replicate tubes and independently extracted using a standard phenol-chloroform procedure [19]. The extracted DNA was quantified on a spectrophotometer and diluted to 50-ng/µl then screened for the presence of four MAC chromosomes and one MIC-limited sequence by PCR and visualization on ethidium bromide stained agarose gels.

### Quantitative PCR

Primers and probes (Table 1) for four MAC chromosomes and one MIC-limited sequence (internal eliminated sequence, IES) were designed using Primer3 primer design software [20] as implemented in Geneious 4.0.4 [21]. The 4 MAC chromosomes carried the genes: *alpha-tubulin* paralog 1 (*α-tubP1*, Genbank accession # AY041123), *alpha-tubulin* paralog 2 (*α-tubP2*, AY041132), the small subunit of *rRNA* (SSU, AF300281), and *elongation factor 1 alpha* (*EF1α*, DQ665311-DQ665312). We chose these chromosomes because we expected these chromosomes to differ in copy number from one another based on previous studies [22]–[24]. The MIC-limited sequence was the third IES in *α-tubP1* (accession # AY330605); this was used as an internal PCR control, as it should only have 2 copies per cell. The efficiency of amplification for all primer pairs was analyzed using DART-PCR [25]. This method allows for direct quantification of amplification efficiency during the experimental real-time quantitative PCR (qPCR). Primers and probes with amplification efficiency below 95% were not used.

**Table 1:** Primers and probes used in qPCR.

Chromosome	Primer		Probe
	Forward	Reverse	
SSU	GATTACGTCCCTGCCCTTTG	TTCACCGGATCACTCAATCG	ACACACCGCCCGTCGCTCCT
*α-tubP1*	CATCTACGATGTTTGCAGAAGACA	CGACGTTGAGGGCACCAT	AACAGACTCATCTCTCAGGTCATCTCTTCGCT
*EF1α*	ACCCAGAAACCAACGAAGTG	TGATCTGCAGGGTGATGAAG	CGTTGGTGGGCAACCTGATG
*α-tubP2*	CATCTACGATGTTTGCAGAAGACA	GCGAGGCTGTCATCGAAGA	CCAACCTGAACAACATCATATCGCGAGTAAC
*atub IES3*	AGAGGTGATGTCGTCCCTAAGG	CATGCGGTCTGTCAAGTACAATC	TGCCGTCGCCACCACTCTACTCCG

**Figure 1:**
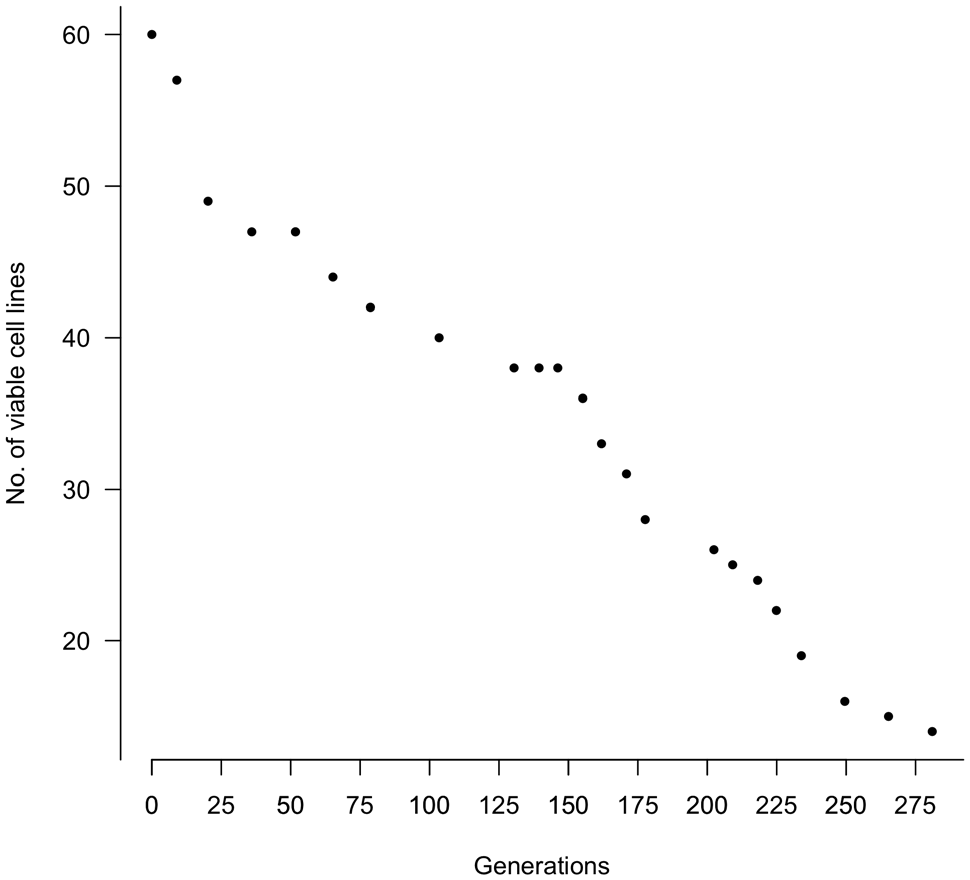
Loss of experimental lineages through 276 rounds of asexual division. Each dot indicates the number of cell lines alive at that generation. The experiment began with 60 cell lines and ended with 14 viable lines.

qPCR was performed in 48-well plates on an ABI StepOne thermal-cycler using Brilliant II qPCR Master Mix with ROX (Stratagene). The reaction program began with 50°C for 2 minutes and a hot start at 95°C for 10 minutes, followed by 50 cycles of 95°C for 15 seconds and 57°C for 30 seconds. Each qPCR reaction was performed in triplicate for each of the independent DNA extractions of the same 6 cell lines at the beginning and end of the experiment. The remaining cell lines either went extinct prior to the end of the experiment (Fig. 1) or lost at least one of the chromosomes being studied.

Changes in chromosome copy number were measured using a standard curve method and a comparative ΔΔCt method [25]–[26]. Our reference sample was *α-tubP1* from an independent strain of *C. uncinata* (ATCC**®** 50194) and our internal control was the native *α-tubP1* IES3. Absolute copy numbers were determined using the reference sample. qPCR was performed on a dilution series of the reference sample that had been ligated into a plasmid, cloned as per manufacture’s instructions (TOPO TA Cloning Kit, Invitrogen), and purified using QIAprep Spin MiniPrep Kit (QIAGEN) along with normally extracted DNA from the reference sample. Running the reference sample in a known dilution series along with its native extraction allows for a calculation of the relationship between qPCR threshold cycle and number of chromosome copies [27]. There were approximately 1345 copies of *α-tubP1* per cell in our reference sample. Comparing the fold change of the target sample qPCR runs with the reference sample copy number gives an estimate of the number of copies of each target chromosome (Dataset S1) [25].

### Chromosome Number Simulation

We modeled the fate of MAC chromosomes under the assumption of no regulation during chromosome segregation with and without stabilizing selection in the R 2.15.1 programming environment (File S1). Simulations were started with a single cell with *x* copies of a given chromosome, where *x* is equal to a randomly selected value chosen from a normal distribution with mean and variance equal to that measured in the six cell lines at the beginning of the experiment. In each replicate, the starting cell went through 12 rounds of asexual division, which is the average number of divisions between transfers in our experimental conditions. At each division, all of the chromosomes were doubled and then distributed to daughter cells assuming stochastic segregation with equal probability of being distributed into either of the daughter cells [14], [28]–[30]. It was assumed that no MAC chromosomes are lost during or between divisions and that generations do not overlap. After 12 asexual divisions, at which time there is a population size of 4096 cells, one cell was randomly chosen to seed the next subculture. This process of subculturing was repeated 23 times.

In the experiment, it is possible for the simulated cell lines to go extinct. To hedge against the loss of a culture in the experimental design, the single-cell transfer method was replicated so that there was a backup culture in case the first culture does not grow. In the simulation, the loss of cell lines occurs when all simulated cells within the culture have a copy number of 0. To mimic the experiment, a second randomly chosen cell is used to seed the simulated backup culture. Each of the backup replicates were run in tandem with main cultures and replenished after each transfer. If a simulated culture becomes extinct, the backup is used, but if both the main and the backup cultures are lost, then that simulated cell line is considered extinct.

To determine the effect of stabilizing selection on copy number and survival during clonal expansion (i.e. the phase of cell growth between single-cell transfer events), we included a selection step based on the following Gaussian fitness function:
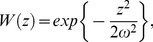
(2)where *ω^2^* determines the strength of stabilizing selection and 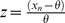 [31]–[32]. Here *θ*, the optimal chromosome copy number, is defined as the starting copy number, and *x_n_* is the chromosome copy number at generation *n*. When *z* is 0, copy number is at its optimum, *W(z)* becomes 1, and the probability of survival is 100%. As *z* moves away from 0, the probability of survival decreases. We varied *ω^2^* to test the effects of varying strengths of selection. For example, as *ω^2^* gets smaller, the selection pressure gets stronger and results in a decrease in the probability of survival. As the copy number at *n* ^th^ generation moves away from the optimal copy number, the probability of survival decreases, allowing fitness to decay in a linear process [31]–[32]. We assumed the optimal chromosome copy number to be the initial copy number as measured in the initial cell lines by qPCR. After each round of division in the simulation, this fitness function is applied to determine the probability of survival of each cell.

To examine chromosome copy number changes, we simulated 6 replicate cell lineages, which is the same number from which we obtained qPCR data under no selection and under various levels of selection 1000 times. After each single-cell transfer, the simulation stores the mean copy number of chromosomes in the 4096 cells. After 276 generations, the change in variance among the 6 replicate lineages was computed between the initial and final time-points (Dataset S1).

To study cell line survival, we simulated 60 cell lineages with a starting copy number representative of the *α-tubP2* chromosome 1000 times. Mortality is defined as the number of lineages that perish due to selection or lost all the chromosomes within that lineage. After each single-cell transfer, the number of extinct cell lines is computed and after 276 generations, the total number of cell lines that survived in the 1000 replicated experiments is recorded.

### Statistical Analysis

To assess which factors affect chromosome copy number in this experiment, we analyzed the data using restricted maximum likelihood (REML) of a linear mixed-model in R with the packages lme4 and languageR [33]–[35]. For each time point, initial and final, we included the chromosome as a fixed effect and cell line as a random effect as well as the interaction between chromosome and cell line in the model. The quantified chromosome copy number was log transformed to account for the magnitude in difference between MAC chromosome copy numbers. P-values from the mixed model were generated by Markov-chain Monte Carlo sampling and considered significant at an alpha of 0.05. Multiple comparisons using the Tukey-Kramer HSD test (α = 0.05) in the R package multcomp were used to determine which chromosomes differed in copy number [36].

The residual among-cell line variance was extracted from the qPCR data using a linear mixed-effect model with REML by the lme4 package in R [33]. The difference in the among-line variance between the initial and final time points was bootstrapped to generate 95% confidence intervals using the boot package in R [37]–[38]. Bootstrapped confidence intervals for the change in the means and for the simulation results were calculated in the same way. If the confidence interval is above 0, then there is an increase in copy number or among-line variance. On the other hand, if the interval is below 0, then there is a decrease in copy number or among-line variance [38].

Pairwise Student t-tests with unequal variances and Bonferroni correction were performed to determine the significance of differences between the experimental data and the simulations, with and without selection. We used this method, rather than relying on overlapping 95% confidence intervals, due to the fact that the confidence interval overlap method is very conservative for Type I errors [39]. P-values were estimated for the Student t-tests by permuting the pairwise comparisons of the bootstrapped variance component of the experimental results with the variance of the replicated simulated results.

## Results

### Senescence of Cell Lines

Sixty cell lineages were created from our original *C. uncinata* culture. Cultures were grown in duplicate in case of cell line loss, but even so, only 14 (23.3%) survived the five months of single-cell transfers (Fig. 1). Surviving cell lines were screened for the presence of all the genes of interest using traditional PCR and gel electrophoresis. Of those still alive at the end of the experiment, only 6 cell lines contained measurable copies of all 4 MAC chromosomes and the micronuclear internal control quantified in this study. None of the remaining 8 cell lines had detectable copies of *α-tubP2* and were not used for further analysis. This observation is consistent with a loss of MAC chromosomes carrying the *α-tubP2* gene. It is unknown why we were unable to detect the MIC copies of *α-tubP2* in these assays, but the absence of detection of the MIC copies of *α-tubP2* could be due to either inadequate template concentration, primer sites not present in the MIC because of gene scrambling or presence of IES at primer sites, or loss of *α-tubP2* from the MIC genome.

### Chromosome Copy Number

**Figure 2:**
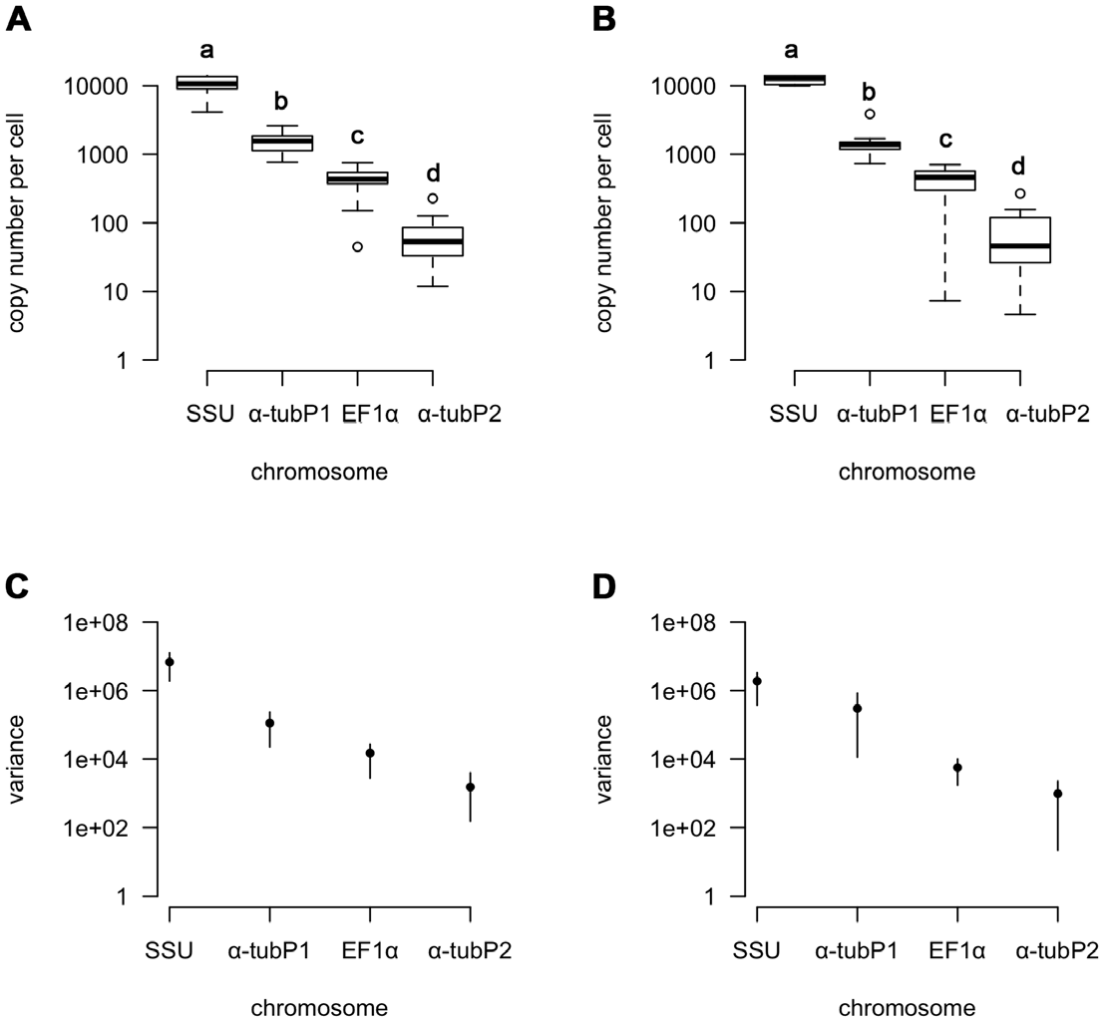
Copy number variation between macronuclear chromosomes. (A) and (C) show the mean copy number and the bootstrapped 95% confidence intervals of the among-line variance component, respectively, for the initial qPCR quantification for SSU, *α-tubP1*, *EF1α*, *α-tubP2*. (B) and (D) show the mean copy number and the bootstrapped 95% confidence intervals of the among-line variance component, respectively, for the final qPCR quantification of the same MAC chromosomes. (A) and (B) (a–d) indicate significant differences between the mean chromosome copy number for each time point (Tukey-HSD, different letters indicate p<0.05).

**Figure 3:**
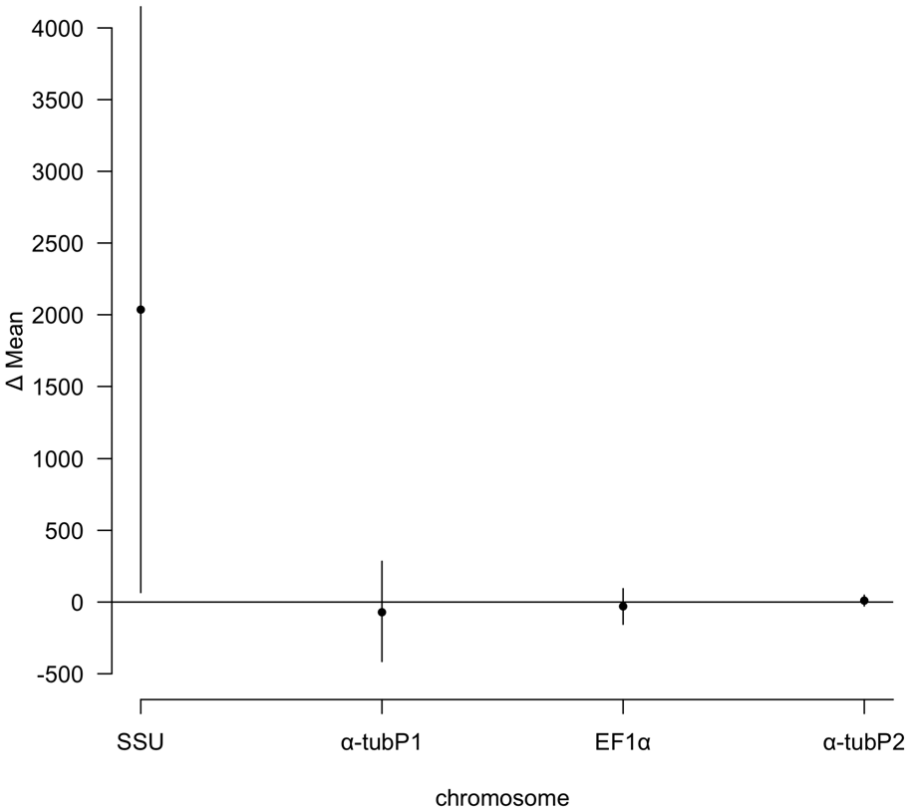
Change in mean chromosome copy number from initial to final time-point. In the *α-tubP1*, *EF1α*, and *α-tubP2* chromosomes, the 95% confidence intervals (error bars) of the change in mean chromosome copy number overlap 0, which indicates there is not a significant difference between the initial and final macronuclear chromosome copy number. SSU has a 95% confidence interval that is greater than 0, meaning that there are more copies of the SSU chromosome at the final time-point than the initial.

The mixed model indicates that the copy number of each chromosome differed significantly from the other chromosomes in the time points that they were measured (MCMC estimated p<0.001). Multiple comparisons using Tukey-HSD indicated that all the chromosomes were significantly different from each other (Fig. 2:A–B). SSU was the most abundant, *α-tubP1* and *EF1α* were at intermediate levels, and *α-tubP2* was present in relatively few copies. To determine whether all of the studied chromosomes significantly increased or decreased in copy number over the course of the experiment, we analyzed each chromosome independently using 95% confidence interval testing. We found that there was no significant change in the mean chromosome copy number between the initial and final time-points for any chromosome except SSU (Fig. 3; bootstrapped 95% CI); SSU showed an increase in chromosome copy number.

### Model of Copy Number Change

**Figure 4:**
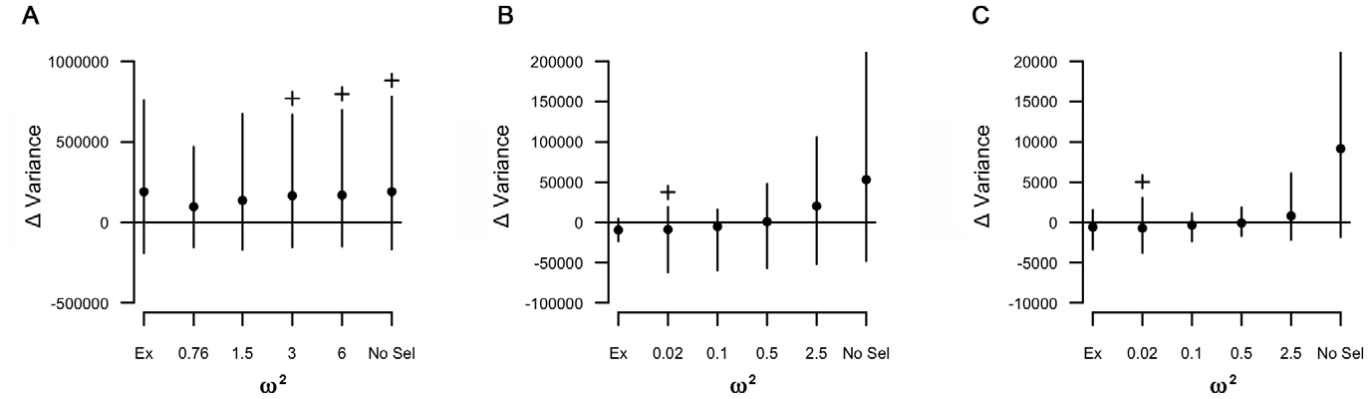
Change in among-line variance of chromosome copy number. The effect of varying levels of stabilizing selection pressure on the change in among-line variance in the simulations and experimental quantifications in the 6 cell lines is shown for chromosomes (A) *α-tubP1*, (B) *EF1α*, and (C) *α-tubP2*. *ω^2^* describes the selection surface. As *ω^2^* increases, the strength of stabilizing selection decreases. ‘Ex’ indicates the experimentally observed among-line variance as estimated with restricted maximum likelihood analysis. ‘No Sel’ represents the simulations without any stabilizing selection pressure. Error bars are the 95% confidence intervals for both the experimentally observed among-line variance and the simulations. Permutated t-tests with unequal variance were conducted between the experimental data (‘Ex’) and the simulations. The cross (+) indicates the values of *ω^2^* at which there is no significant difference between the change in variance of the experimental results and the change in variance of the simulation.

A simulation was performed to determine whether our experimental results are consistent with stochastic segregation of chromosomes during asexual division. The model predicts no change in the mean copy number of any chromosome, which is what we observed for all chromosomes except the one carrying SSU (Fig. 3). For the genes whose mean chromosome copy number did not change over the course of the experiment (i.e. all but SSU), we tested whether the among-line variance results could be explained by stabilizing selection on chromosome copy number. We found that *α-tubP1* did not show any difference in the mean change in variance between the experimental and the simulated cell lines for low levels of selection to no selection pressure. Adding stabilizing selection to the simulation does result in a closer fit between the experimental results and the simulated data for *EF1α* and *α-tubP*2 (Fig. 4).

**Figure 5:**
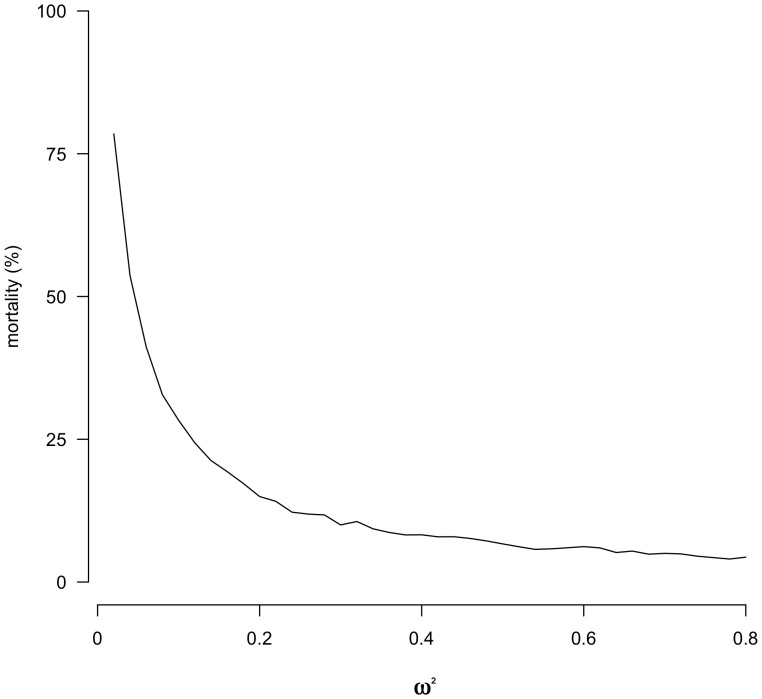
Cell line death rate under varying levels of stabilizing selection. One thousand replicates of 60 cell lines were simulated with values of *ω^2^* ranging from 0.02 to 0.80 in intervals of 0.02. Mortality occurs when all the cells within a lineage having either perished due to selection pressure or having not received at least 1 MAC chromosome. Shown are the results of a simulation with a starting chromosome copy number of 66, which was the copy number value of the chromosome with the lowest copy number in this study. The simulation followed the model of random segregation of macronuclear chromosomes with stabilizing selection acting on changes from the optimal (initial) chromosome copy number. *ω^2^* determines the strength of stabilizing selection and as *ω^2^* decreases, stabilizing selection increases and results in increased cell mortality.

Increasing selection pressure in the model also increased the mortality of the cells in the population (Fig. 5). The percent mortality of 1000 replicates of 60 cell lines were measured after putting the cell lines throught the simulation at different levels of *ω^2^* (0.02 to 0.8), Eq (2). At *ω^2^*≤0.4, there is a sharp increase in the mortality rate.

## Discussion

Like other ciliates with gene-size chromosomes [10], [40], we find that *C. uncinata* chromosomes vary in copy number (Fig. 2). The observed differences in copy number could be due to either independent control of amplification of each chromosome during development of the MAC or accumulated differences due to lack of regulation during segregation. The fact that our results correspond well with previous estimates of chromosome copy number in this species [41]–[43] suggests that the overall differences between chromosomes are due to differential amplification. Likewise, as expected based on the high copy number of the rDNA-containing chromosome in other ciliates, we also find this chromosome at a high copy number, supporting the role of differential chromosome amplification during development [22], [44].

To assess whether these differentially amplified chromosomes are randomly distributed among daughter cells during asexual division, we performed an experiment similar to mutation accumulation experiments (reviewed in [45]). We allowed cell lineages to undergo asexual division for an estimated 276 generations with periodic bottlenecking to moderate the effects of selection. We find no change in the mean chromosome copy number in three of the four quantified chromosomes. This is expected if there is no selection or mutational pressure to change copy number, which is consistent with our simulation.

The rDNA-carrying chromosome was the only chromosome to experience an increase in copy number in four out of six cell lines (Fig. 3), which is consistent with the action of selection to increase copy number of this chromosome. rDNA genes are some of the most highly conserved genes and are found at high, and often variable, copy number throughout eukaryotes [46]–[47]. Experimental manipulation of rDNA copy number in other systems supports a role of selection in maintaining this locus at high copy number [48]–[49].

In contrast to most mutation accumulation experiments and the predictions of our simulation, the among-line variance in copy number increased less than expected for all chromosomes except *α-tubP1* (Fig. 2:C–D and Fig. 4). This suggests either that strong stabilizing selection (or directional selection, in the case of SSU) is acting on the cells, limiting the change in chromosome copy number, or the action of an as yet unknown regulatory mechanism to control chromosome copy number.

Comparison of our cell line senescence results (Fig. 1) with the model of Duerr et al. [14] suggests a lack of regulation of chromosome segregation. Under the Duerr et al. [14] model of regulated segregation, we would expect a much higher level of viability in our cell lines. For example, it would take about 2000 generations for a chromosome with 14 copies to be lost under the regulatory model [14]. If the cause of cell line death in our experiment is due to chromosomal loss [50], then it is highly unlikely that *C. uncinata* is able to regulate its MAC chromosome copy number during asexual division. Initial chromosome copy number of the four quantified chromosomes range from approximately 60 to 10,000 copies, yet over 75% of the lineages became extinct. Under this regulatory model, it would be expected that *C. uncinata* would be viable much longer than that observed.

We tested the possibility that stabilizing selection could explain the observed mortality and contribute to the smaller than expected change in variance in chromosome copy number. We modeled the effect of selection favoring the initial copy number for each chromosome in our experiment. We find that varying levels of stabilizing selection are sufficient to explain the observed change in variance in chromosome copy number among lines over the course of the experiment (Fig. 4). We also find that stabilizing selection may account for our observed high level of cell line loss. As the strength of stabilizing selection increases, the mortality of cell lines also increases (Fig. 5).

Previous studies suggest that chromosome copy number in ciliates with gene-size chromosomes is correlated with mRNA expression level [24], [40]. Thus, we would expect arbitrary changes in chromosome number to have deleterious fitness consequences due to transcription levels higher or lower than the optimum. Bellec and Katz [43], however, report a lack of correlation in *C. uncinata* between chromosome copy number and gene expression level, including one of the genes studied here (*α-tubP1*). Thus, the exact mechanism that could result in stabilizing selection waits further testing. In addition, while we assumed that all of the genes studied are essential for cell function, experimental tests of this assumption would provide additional insight into possible mechanisms of selection. Our study therefore suggests that selection is likely to play a role in controlling chromosome copy number during asexual division, but the mechanism of selection is unclear. Nonetheless, these results also leave open the possibility of other regulatory mechanisms controlling chromosome copy number in *C. uncinata*.

## Supporting Information

File S1 **Macronuclear chromosome distribution simulator.** R script containing a set of functions to generate the simulated distribution of macronuclear chromosomes during asexual division in *Chilodonella uncinata.*
(R) Click here for additional data file.

Dataset S1 **Chromosome copy numbers from the experimental and simulated cell lines of Dataset of** ***Chilodonella uncinata*** **.** dataset.zip is a compressed file. exp_data.csv contains the experimental data derived from qPCR analyses (Cell.line = original cell line; Time = time point of the DNA extraction; DNA.extract = DNA extract replicate; Gene = macronuclear chromosome; CNPC = copy number of the macronuclear chromosome per cell). The remainder of the CSV files are from the simulations. Each CSV file contains data on 1000 simulations where the change in variance from initial to final for each simulation is stored under column ‘x’. See _readme.txt in the compressed file dataset.zip for documentation of the file names.
(ZIP)

## References

[1] Redon R, Ishikawa S, Fitch KR, Feuk L, Perry GH, et al (2006) Global variation in copy number in the human genome. Nature 444: 444–454. 17122850 10.1038/nature05329 PMC2669898

[2] Locke DP, Sharp AJ, McCarroll SA, McGrath SD, Newman TL, et al (2006) Linkage disequilibrium and heritability of CNPs within duplicated regions of the human genome. Am J Hum Genet 79: 275–290. 16826518 10.1086/505653 PMC1559496

[3] Stankiewicz P, Lupski JR (2010) Structural variation in the human genome and its role in disease. Annu Rev Med 661: 437–455. 10.1146/annurev-med-100708-204735 20059347

[4] Kidd JM, Cooper GM, Donahue WF, Hayden HS, Sampas N, et al (2008) Mapping and sequencing of structural variation from eight human genomes. Nature 453: 56–64. 18451855 10.1038/nature06862 PMC2424287

[5] Gonzales E, Kulkarni H, Bolivar H, Mangano A, Sanchez R, et al (2005) The influence of CCL3L1 gene-containing segmental duplications of HIV-1/AIDS susceptibility. Science 307: 1434–1440. 15637236 10.1126/science.1101160

[6] Aitman TJ, Dong R, Vyse TJ, Norsworthy PJ, Johnson MD, et al (2006) Copy number polymorphism in *Fcgr3* predisposes to glomerulonephritis in rats and humans. Nature 439: 851–855. 16482158 10.1038/nature04489

[7] Auer H (2008) Expression divergence and copy number variation in the human genome. Cytogenet Genome Res 128: 278–282. 10.1159/000184718 19287165

[8] Infante JJ, Dombek KM, Rebordinos L, Cantoral JM, Young ET (2003) Genome-wide amplifications caused by chromosomal rearrangements play a major role in the adaptive evolution of natural yeasts. Genetics 165: 1745–1759. 14704163 10.1093/genetics/165.4.1745 PMC1462916

[9] Force A, Lynch M, Pickett F, Amoresa A, Yana Y, et al (1999) Preservation of duplicated genes by complementary, degenerative mutations. Genetics 151: 1531–1545. 10101175 10.1093/genetics/151.4.1531 PMC1460548

[10] Prescott DM (1994) The DNA of ciliated protozoa. Microbiol Rev 58: 233–267. 8078435 10.1128/mr.58.2.233-267.1994 PMC372963

[11] Katz LA (2001) Evolution of nuclear dualism in ciliates: a reanalysis in light of recent molecular data. J Syst Evol Microbiol 51: 1597–1592. 10.1099/00207713-51-4-1587 11491362

[12] Zufall RA, Katz LA (2007) Micronuclear and macronuclear forms of β-tubulin genes in the ciliate *Chilodonella uncinata* reveals insights into genome processing and protein evolution. J Eukaryot Microbiol 54: 275–282. 17552983 10.1111/j.1550-7408.2007.00267.x

[13] Nowacki M, Haye JE, Fang W, Vijayan V, Landweber LF (2010) RNA-mediated epigenetic regulation of DNA copy number. Proc Natl Acad Sci U S A 107: 21140–22144. 21078984 10.1073/pnas.1012236107 PMC3009799

[14] Duerr HP, Eichner M, Ammermann D (2004) Modeling senescence in hypotrichous ciliates. Protist 155: 45–52. 15144057 10.1078/1434461000163

[15] Doerder FP, Deak JC, Lief JH (1992) Rate of phenotypic assortment in Tetrahymena thermophile. Dev Genet 13: 126–132. 1499154 10.1002/dvg.1020130206

[16] Eisen JA, Coyne RS, Wu Martin, Wu Dongying, Thiagarajan M, et al (2006) Macronuclear genome sequence of the ciliate *Tetrahymena thermophila,* a model eukaryote. PLoS Biol 4: e286 doi: 10.1371/journal.pbio.0040286. 16933976 10.1371/journal.pbio.0040286 PMC1557398

[17] Baird SE, Klobutcher LA (1991) Differential DNA amplification and copy number control in the hypotrichous ciliate *Eupotes crassus* . J Protozool 38: 136–140. 1902260 10.1111/j.1550-7408.1991.tb06033.x

[18] Robinson T, Katz LA (2007) Non-Mendelian inheritance of two cytoskeletal genes in the ciliate *Chilodonella uncinata* . Mol Biol Evol 24: 2495–2503. 17890762 10.1093/molbev/msm203

[19] Ausubel FM, Brent R, Kingston RE, Moore DD, Seidman JG, et al.. (1993) Current Protocols in Molecular Biology, Wiley-Liss, New York.

[20] Rozen S, Skaletsky HJ (2000) Primer3 on the WWW for general users and for biologist programmers. In: Krawetz S, Misener S, editors. Bioinformatics Methods and Protocols: Methods in Molecular Biology. Totowa: Humana Press. 365–386. Available: http://primer3.sourceforge.net. Accessed 2013 Jan 15. 10.1385/1-59259-192-2:365 10547847

[21] Drummond AJ, Ashton B, Buxton S, Cheung M, Cooper A, et al.. (2008) Geneious v4.0.4. Available: http://www.geneious.com. Accessed 2013 Jan 15.

[22] Orias E, Bradshaw AD (1992) Stochastic developmental variation in the ratio of allelic rDNAs among newly differentiated, heterozygous macronuclei of *Tetrahymena thermophila* . Dev Genet 13: 87–93. 1395146 10.1002/dvg.1020130114

[23] Blackburn EH, Karrer KM (1986) Genomic reorganization in ciliated protozoans. Ann Rev Genet 20: 501–521. 3101582 10.1146/annurev.ge.20.120186.002441

[24] La Terza A, Miceli C, Luporini P (1995) Differential amplification of pheromone genes of the ciliate *Euplotes raikovi* . Dev Genet 17: 272–279. 8565333 10.1002/dvg.1020170312

[25] Peirson SN, Butler JN, Foster R (2003) Experimental validation of novel and conventional approaches to quantitative real-time PCR data analysis. Nucleic Acids Res 31: e73. 12853650 10.1093/nar/gng073 PMC167648

[26] Ding C, Cantor CR (2004) Quantitative analysis of nucleic acids – the last few years of progress. J Biochem Mol Biol 37: 1–10. 14761298 10.5483/bmbrep.2004.37.1.001

[27] Adams PS (2006) Data Analysis and Reporting. In: Dorak MT, editors. Real-Time PCR. New York: Taylor & Francis Group. 40–44.

[28] Kimura M (1957) Some problems of stochastic processes in genetics. Ann Appl Stat 28: 882–901.

[29] Schensted IV (1958) Model of subnuclear segregation in the macronucleus of ciliates. Am Nat 92: 161–170.

[30] Preer JR (1976) Quantitative predictions of random segregation models of the ciliate macronucleus. Genet Res 27: 227–238. 1278685 10.1017/s0016672300016426

[31] Wagner GP, Booth G, Bagheri-Chaichian H (1997) A population genetic theory of canalization. Evolution 51: 329–347. 28565347 10.1111/j.1558-5646.1997.tb02420.x

[32] Roff DA (2010) Genetic Models. In: Modeling Evolution: An Introduction to Numerical Methods. New York: Oxford University Press. 223–269.

[33] Bates D, Mächler M, Bolker B (2011) lme4: Linear mixed-effects models using S4 classes. R package version 0.999375-42.

[34] Baayen RH (2011) language: Data sets and functions with “Analyzing Linguistic Data: A practical introduction to statistics” R package version 1.4.

[35] Baayen RH, Davidson DJ, Bates DM (2008) Mixed-effects modeling with crossed random effects for subjects and items. J Mem Lang 59: 390–412.

[36] Hothorn T, Bretz F, Westfall P (2008) Simultaneous inference in general parametric models. Biom J 50: 346–353. 18481363 10.1002/bimj.200810425

[37] Canty A, Ripley B (2012) boot: Bootstrap R (S-Plus) function. R package version 1.3–5.

[38] Davison AC, Hinkley DV (1997) Bootstrap Methods and their Applications. Cambridge: Cambridge University Press.

[39] Schenker N, Gentleman JF (2001) On judging the significance of differences by examining the overlap between confidence intervals. Am Stat 55: 182–186.

[40] Xu K, Doak TG, Lipps HJ, Wang JM, Swart EC, Chang WJ (2012) Copy number variatons of 11 macronuclear chromosomes and their gene expression in *Oxytricha trifallax* . Gene 505: 75–80. 22669045 10.1016/j.gene.2012.05.045

[41] Israel RL, Kosakovsky Pond SL, Muse SV, Katz LA (2002) Evolution of duplicated alpha-tubulin genes in ciliates. Evolution 56: 1110–1122. 12144013 10.1111/j.0014-3820.2002.tb01425.x

[42] Katz LA, Lasek-Nesselquist E, Snoeyenbos-West OLO (2003) Structure of the micronuclear α-tubulin gene in the phyllopharyngean ciliate *Chilodonella uncinata*: implications for the evolution of chromosomal processing. Gene 315: 15–19. 14557060 10.1016/j.gene.2003.08.003

[43] Bellec L, Katz LA (2012) Analyses of chromosome copy number and expression level of four genes in the ciliate *Chilodonella uncinata* reveal a complex pattern that suggests epigenetic regulation. Gene 504: 303–308. 22588027 10.1016/j.gene.2012.04.067 PMC3412129

[44] Kaufmann J, Klein A (1992) Gene dosage as a possible major determinant for equal expression levels of genes encoding RNA polymerase subunits in the hypotrichous ciliate *Euplotes octocarinatus* . Nucleic Acids Res 20: 4445–4450. 1408746 10.1093/nar/20.17.4445 PMC334170

[45] Halligan DL, Keightley PD (2009) Spontaneous mutation accumulation studies in evolutionary genetics. Annu Rev Ecol Evol Syst 40: 151–172.

[46] Long EO, David IB (1980) Repeated genes in eukaryotes. Ann Rev Biochem 49: 727–764. 6996571 10.1146/annurev.bi.49.070180.003455

[47] Eickbush TH, Eickbush DG (2007) Finely orchestrated movements: evolution of the ribosomal RNA genes. Genetics 175: 477–485. 17322354 10.1534/genetics.107.071399 PMC1800602

[48] Kobayashi T, Heck DJ, Nomura M, Horiuchi T (1998) Expansion and contraction of ribosomal DNA repeats in *Saccharomyces cerevisiae*: requirement of replication fork blocking (Fob1) protein and the role of RNA polymerase I. Genes Dev. 12: 3821–3830. 10.1101/gad.12.24.3821 PMC317266 9869636

[49] Ide S, Miyazaki T, Maki H, Kobayashi T (2010) Abundance of ribosomal RNA gene copies maintains genome integrity. Science 327: 693–696. 20133573 10.1126/science.1179044

[50] Bell G (1988) Sex and Death in Protozoa: The History of Obsession. Cambridge: Cambridge University Press.

